# Whole brain radiotherapy after local treatment of brain metastases in melanoma patients - a randomised phase III trial

**DOI:** 10.1186/1471-2407-11-142

**Published:** 2011-04-17

**Authors:** Gerald Fogarty, Rachael L Morton, Janette Vardy, Anna K Nowak, Catherine Mandel, Peta M Forder, Angela Hong, George Hruby, Bryan Burmeister, Brindha Shivalingam, Haryana Dhillon, John F Thompson

**Affiliations:** 1 St Vincent's and Mater Hospitals, Radiation Oncology, University of New South Wales, Randwick, Australia; 2 School of Public Health, Sydney Medical School, The University of Sydney, Sydney, Australia; 3 Australia and New Zealand Melanoma Trials Group (ANZMTG), Poche Centre, North Sydney, Australia; 4 Sydney Cancer Centre, Sydney, Australia; 5 Central Clinical School, Sydney Medical School, The University of Sydney, Sydney, Australia; 6 School of Medicine and Pharmacology, Sir Charles Gairdner Hospital, Nedlands, Australia; 7 Peter MacCallum Cancer Centre East Melbourne & University of Melbourne, Parkville, Australia; 8 Research Centre for Gender Health and Aging, The University of Newcastle, Newcastle, Australia; 9 NHMRC Clinical Trials Centre, The University of Sydney, Sydney, Australia; 10 Melanoma Institute Australia, North Sydney, Australia; 11 Princess Alexandra Hospital, Brisbane, Australia; 12 Trans-Tasman Radiation Oncology Group (TROG), Australia; 13 Centre for Medical Psychology & Evidence-based Decision-making, Sydney Medical School, The University of Sydney, Australia

## Abstract

**Background:**

Cerebral metastases are a common cause of death in patients with melanoma. Systemic drug treatment of these metastases is rarely effective, and where possible surgical resection and/or stereotactic radiosurgery (SRS) are the preferred treatment options. Treatment with adjuvant whole brain radiotherapy (WBRT) following neurosurgery and/or SRS is controversial. Proponents of WBRT report prolongation of intracranial control with reduced neurological events and better palliation. Opponents state melanoma is radioresistant; that WBRT yields no survival benefit and may impair neurocognitive function. These opinions are based largely on studies in other tumour types in which assessment of neurocognitive function has been incomplete.

**Methods/Design:**

This trial is an international, prospective multi-centre, open-label, phase III randomised controlled trial comparing WBRT to observation following local treatment of intracranial melanoma metastases with surgery and/or SRS. Patients aged 18 years or older with 1-3 brain metastases excised and/or stereotactically irradiated and an ECOG status of 0-2 are eligible. Patients with leptomeningeal disease, or who have had previous WBRT or localised treatment for brain metastases are ineligible. WBRT prescription is at least 30 Gy in 10 fractions commenced within 8 weeks of surgery and/or SRS. Randomisation is stratified by the number of cerebral metastases, presence or absence of extracranial disease, treatment centre, sex, radiotherapy dose and patient age. The primary endpoint is the proportion of patients with distant intracranial failure as determined by MRI assessment at 12 months. Secondary end points include: survival, quality of life, performance status and neurocognitive function.

**Discussion:**

Accrual to previous trials for patients with brain metastases has been difficult, mainly due to referral bias for or against WBRT. This trial should provide the evidence that is currently lacking in treatment decision-making for patients with melanoma brain metastases. The trial is conducted by the Australia and New Zealand Melanoma Trials Group (ANZMTG-study 01-07), and the Trans Tasman Radiation Oncology Group (TROG) but international participation is encouraged. Twelve sites are open to date with 43 patients randomised as of the 31st March 2011. The target accrual is 200 patients.

**Trial registration:**

Australia and New Zealand Clinical Trials Register (ANZCTR): ACTRN12607000512426

## Background

The incidence of central nervous system (CNS) metastases in patients with metastatic melanoma ranges from 10% to 40% in clinical studies[1] and is even higher in autopsy series, with as many as 72% of patients with metastatic melanoma having CNS involvement [2]. Local control of brain metastases from melanoma is a significant problem. Sampson et al. reported an overall median survival time of 3.8 months in 702 patients with clinically significant melanoma brain metastases, treated with either palliative chemotherapy, radiotherapy or surgery [3]. These metastases contributed to the death of 94.5% of these patients.

As well as seeking to prolong survival, maintaining neurocognitive function in these patients is pivotal to their quality of life. The clinically apparent metastases can often be treated locally by neurosurgery and/or stereotactic radiosurgery (SRS), with the option of post-operative whole brain radiotherapy (WBRT). The objective of WBRT in this setting is to treat clinically undetectable micrometastases elsewhere in the brain; these are considered likely to be present in many patients and, if not controlled, will later manifest as distant intracranial treatment failure. However the role of WBRT after local treatment is controversial. Proponents say that WBRT to prevent or delay intra-cranial disease recurrence provides worthwhile palliation. Opponents argue against WBRT as a survival benefit has never been demonstrated in this situation and there is a risk of neurotoxicity. These opinions are based on studies in other malignancies, predominantly non small-cell lung cancer and lymphoma, and retrospective analyses [4-7]. There have been no randomised clinical trials (RCTs) for this specific scenario in patients with metastatic melanoma. A complicating factor is that there exists a strong anecdotal impression among some clinicians that melanoma is a uniformly radioresistant tumour, although there is no level 1 clinical evidence for this. As a result, current clinical practice varies widely, with some units actively encouraging WBRT while others rarely or never offer it.

In a randomised controlled trial of 95 patients with a variety of solid tumour types who received WBRT following surgical excision of brain metastases, those receiving WBRT (50.4 Gy over 5 weeks) had a 52% reduction in intracranial recurrence and a 30% reduction in death from neurological causes compared to those randomised to observation [4]. Although the time to intracranial recurrence was substantially longer in the WBRT group (220 weeks versus 26 weeks, p = < 0.001), the median overall survival was not significantly different between the two groups (11 months versus 10 months, p = 0.39), probably because a high proportion of patients in both groups had extra-cranial disease.

A second randomised controlled trial comparing SRS plus WBRT (30 Gy in 10 fractions) versus SRS alone in 132 patients with 1-4 brain metastases reported a 30% reduction in local intracranial recurrence and a 22% reduction in distant intracranial recurrence at 12 months in the WBRT group (p = 0.001) [6]. There was no difference between the groups in overall survival (7.5 months versus 8 months) or neurological death. In a subset of patients neurological toxicity and neurological function were assessed radiologically and using the Karnofsky Performance Scale (KPS) and the Mini Mental State Examination (MMSE). There were no significant differences in toxicity or function.

A more recent randomised trial undertaken by the European Organisation for Research and Treatment of Cancer (EORTC) comparing adjuvant WBRT (30 Gy in 10 fractions) to observation after surgery or radiosurgery of 1-3 brain metastases (predominantly from lung, breast and kidney cancer) assessed the time to functional decline (performance score > 2) [8]. Overall survival was similar in the WBRT and observation arms (median 10.9 versus 10.7 months), however WBRT reduced the 2-year relapse rate both at both initial sites (surgery: 59% to 27%, p = 0.001; SRS: 31% to 19%, p = 0.040) and at new sites (surgery: 42% to 23%, p = 0.008; SRS 48% to 33%, p = 0.023).

There has been some recent controversy about whether WBRT affects neurocognitive function [9,10]. Assessment of neurocognitive function and health-related quality of life (HRQOL) are essential for the interpretation of WBRT in the context of the patient's experience. It is clear that tools such as the MMSE, whilst appealing in their simplicity, were developed to screen for dementia and show poor sensitivity in detecting cognitive impairment in patients with brain tumours [11]. The ideal tool should be brief, simple, sensitive, inexpensive and, if administered repeatedly, should have alternative versions to reduce the effects of learning [12]. A battery of tests which fit these criteria (including Hopkins Verbal Learning Test-Revised, Controlled Oral Word Association of the Multilingual Aphasia Examination, Trail Making Test, Stroop Color-Word Test and the Digit Span test) have been shown to be feasible in previous clinical trials in patients with brain metastases [13].

The European Organization for Research and Treatment of Cancer Quality of Life Questionnaire (EORTC QLQ-C30) has been validated in numerous malignancies including neurological malignancies [14,15] and measures five functional domains as well as global quality of life and symptoms of fatigue, nausea, pain, dyspnoea, insomnia, appetite loss, constipation, diarrhoea, and financial difficulties. The EORTC BN-20 is a brain cancer-specific module that measures four multi-item domains (future uncertainty, visual disorder, communication deficit, and motor dysfunction) [15]. A change in scores of ≥ 10 on a scale of 0-100 persisting for 4 or more weeks has been shown to represent a clinically meaningful and subjectively significant change that is associated with change in disease status [15]. These tools are considered appropriate, validated and sensitive in this population.

Using magnetic resonance imaging (MRI) as the primary means of assessment, the current trial will investigate whether WBRT following complete local treatment of intracranial melanoma metastases improves distant intracranial control and hence demonstrate whether radiotherapy in this scenario can control microscopic intracerebral melanoma. If so, then it may contribute to more clinically meaningful outcomes such as increased time to functional decline from further intracranial disease or neurological death, without causing excessive neurotoxicity. Trial recruitment will be supported through referrals from multi-disciplinary melanoma teams. The trial will be offered to all eligible patients in whom intracranial disease control has been obtained by complete surgical resection and/or SRS.

### Objectives

**Figure 1:**
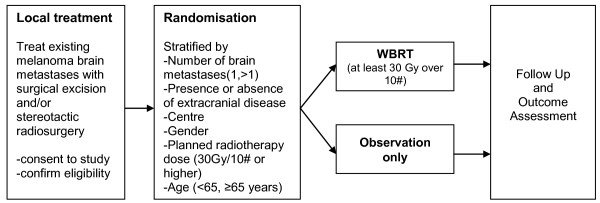
**Trial schema**.

This study aims to assess the value of treating brain metastases in patients with AJCC stage IV melanoma using adjuvant post-operative WBRT in the hope of improving disease control, and quality of life, while maintaining satisfactory cognitive performance. The primary objective is to assess the effect of WBRT (after localised treatment for melanoma brain metastases) on distant intracranial control, as assessed by MRI scanning. (Figure 1) Distant intracranial control is defined as control within the brain 1 cm or more from a previous metastasis. The primary hypothesis is that as a result of whole brain radiotherapy, there will be a 20% reduction in the rate (proportion) of distant intracranial metastases after at least 12 months of follow-up, compared to the control (observation only) arm.

The secondary objectives are to assess the effect of WBRT on:

1. Time to intracranial failure (local, distant and overall (local+ distant)) as assessed by MRI

2. Quality of life

3. Performance status

4. Neurocognitive function

5. Overall survival

6. Death from neurological causes

Deterioration in neurocognitive function and distant intracranial failure will be assessed for the following subgroups: one versus more than one treated cerebral metastasis; presence of extracranial disease versus none; < 65 years of age versus ≥65 years of age.

## Methods/Design

### Trial Design

This trial is an international multi-centre, open-label, stratified, 2-arm randomised phase III trial. Patients will be randomised 1:1 to WBRT or observation. The trial has been approved by the Cancer Institute NSW Clinical Research Ethics Committee #2007C/11/032 and relevant hospital ethics committees in each participating centre.

### Participants

Patients will be recruited mainly from participating multi-disciplinary melanoma treatment centres. Patients will be identified through routine scanning showing asymptomatic metastases or by investigation of intracranial symptoms. The eligibility criteria are listed in Table 1.

**Table 1:** Eligibility criteria - inclusions and exclusions

Inclusion criteria	Exclusion criteria
• One to three (1-3) intracranial metastases on MRI from melanoma, all locally treated with either surgical excision and/or stereotactic irradiation. (It will be assumed that the metastases are melanoma if the patient has documented histological or radiological concurrent extracranial disease that has already categorised the patient as stage IV). If the cerebral lesion(s) is/are the first presentation of stage IV disease, then one metastasis must be histologically proven to be melanoma for the patient to be included in the study. • Life expectancy of at least 6 months. • Aged 18 years or older. • WBRT must begin within 8 weeks of completion of local treatment and within 4 weeks of randomisation. • Able to have an MRI brain scan with contrast enhancement. Estimated glomerular filtration rate (eGFR) is adequate at the discretion of the radiologist and capable of having gadolinium-containing contrast medium for MRI (as per practice guidelines). • Localised treatment of all brain metastases no more than 6 weeks prior to randomisation. • An ECOG performance status of 0, 1 or 2 at randomisation. • CT scan of chest, abdomen and pelvis within 12 weeks of randomisation. • Serum lactate dehydrogenase (LDH) must be ≤ 2 times upper limit of the participating centre's reference range. • Able to provide written informed consent.	• Any untreated intracranial disease. • Any previous intracranial treatment (surgical excision and/or stereotactic irradiation treatment and/or WBRT) prior to this diagnosis of intracranial melanoma. • Evidence of leptomeningeal disease on pre-local treatment MRI scan. • Patients with prior cancers, except: those diagnosed more than five years ago with no evidence of disease recurrence within this time; successfully treated basal cell and squamous cell skin carcinoma; or carcinoma in-situ of the cervix. • A medical or psychiatric condition that compromises ability to give informed consent or complete the protocol. • Positive urine pregnancy test for women of childbearing potential.

In order to be eligible, patients must meet all of the specified inclusion and exclusion criteria. In addition, patients will be excluded from the neurocognitive function (NCF) and quality of life aspects of the study if their fluency of oral and written English is less than Year 8 standard. Centres in countries where English is not a main language can still participate in the primary endpoint of the study but not the NCF component.

#### Setting

Melanoma treatment centres and tertiary cancer hospitals with facilities for WBRT.

### Interventions

#### Neurosurgery for melanoma brain metastases

Neurosurgery will be conducted according to the usual practice at the treating centre. A lesion will be deemed to be completely excised if the treating neurosurgeon reports complete excision. A patient with a lesion not completely excised should be referred for SRS Incomplete excision is not an exclusion criterion; patients whose lesions are not completely excised may still be randomised into the trial to receive WBRT or observation. Histopathology reports using hematoxylin and eosin stains as well as immunostains (at least one of S100 or HMB45) are required.

#### Stereotactic radiosurgery (SRS) of melanoma brain metastases

SRS may be given to the target lesion(s) definitively or to the surgical cavity(ies) post resection. It should be given according to the usual practice at the treating centre. Full records of the procedure need to be included in the written SRS report. For quality assurance purposes, copies of the prescription page and computer dosimetry on axial, coronal and sagittal planning CT images through the target(s) are required.

#### Control intervention

Observation only (no WBRT) with regular assessment of outcomes at the same time points specified for the intervention arm.

### Assessment of Outcome

The primary endpoint of the study will be the proportion of patients with distant intracranial failure (as determined through MRI assessment) at 12 months. Distant intracranial failure is defined as new lesions appearing 1 cm or more from a previous index metastasis. New onset leptomeningeal disease after the randomisation MRI will be recorded as new distant intracranial disease. Patients who fail locally only will not be considered as having an event and their time will be measured from randomisation to their last known follow-up date. Patients progressing or dying from extracranial disease or other causes will be considered as having a competing risk for distant intracranial failure.

The secondary endpoints include:

(i) Time to distant intracranial failure as determined by MRI. This is defined as the time from the date of randomisation to recurrence of disease at a distance of 1 cm or more from previously treated metastases. In the absence of distant intracranial failure (i.e. for patients censored before intracranial failure could be observed), time to failure will be measured from randomisation to date of last known contact (i.e. censoring time).

(ii) Time to local intracranial failure as determined by MRI. This is defined as the time from the date of randomisation to recurrence of disease within 1 cm of previously treated metastases.

(iii) Time to overall (distant + local) intracranial failure as determined by MRI.

(iv) Deterioration in neurocognitive function (NCF). This is measured by a battery of assessments including Hopkins Verbal Learning Test, Controlled Oral Word Association Test, Trail Making Test Part A and B, Stroop Color-Word Test (Adult Version), Digit Span (Forwards and Backwards). The proportion of patients completing neurocognitive function assessments at the baseline visit and at each 2-monthly follow up visit will be determined for each of the treatment groups (WBRT and Observation) together with a descriptive summary of Global Deficit Scores (GDS) scores. The main neurocognitive function endpoint will be defined as the proportion of patients who have deteriorated from the baseline visit at any time in the study period by at least 0.3 units on the GDS scale.

(v) Time to deterioration in health related quality of life. This is measured by EORTC QLQ-C30 with Brain module (EORTC BN-20) questionnaires. The completion rates for quality of life questionnaires at the baseline visit and at each 2-monthly follow-up visit will be determined for each treatment group together with descriptive summaries of scores. The primary QOL endpoint will be time to deterioration in role function from randomisation, with deterioration defined as a decrease of ≥ 10 points on a 0-100 scale persisting for at least 4 weeks. Secondary endpoints will be time to deterioration in global QOL, drowsiness, communication difficulties, motor dysfunction and social function items/domains.

(vi) Time to deterioration in performance status as measured by ECOG criteria. This is defined as the time that elapses between randomisation and the first recorded worsening (increase) in ECOG performance status.

(vii) Overall survival. This will include time to death due to any cause; time to death due to a neurological cause; cause of death (cancer related or not); and cause of death if cancer related, due to neurological progression or not. Overall survival will be assessed from date of randomisation to date of death from any cause. Patients remaining alive or lost to follow-up will be censored at the date of last known contact.

### Sample size

It has been assumed on the basis of previous studies[4,6] that the proportion of patients having distant intracranial metastases at 12 months post-randomisation will be 55% in the observation arm (surgery and/or SRS only) and 33% in the WBRT arm. With 200 patients and assuming 10% non-adherence, this study will have 80% power to detect an absolute risk reduction of 22% at the 5% significance level (two-tailed). To achieve a total sample of 200 patients, it is assumed that patients will be accrued over five years. This assumes a uniform accrual rate of 40 patients each year, which will require participation from international centres as well as centres in Australia and New Zealand.

#### Interim analyses and stopping guidelines

An independent data safety monitoring committee (DSMC) will regularly monitor the occurrence of serious clinical events. One formal efficacy analysis will be performed after 45 events have been observed (the expected number of events after 100 randomised patients have completed 12 months of follow-up). The events used for this formal interim analysis will be distant intracranial failure after 12 months of follow-up. The stopping rule for the study on the basis of efficacy will be a nominal significance level of p = 0.003 (3 standard deviations) to maintain an overall Type I error probability of 5%.

The DSMC will monitor the trial for safety outcomes including unacceptable acute radiotherapy toxicity (any Grade 4 toxicity); accrual less than 30% of the expected number of patients within the first 36 months; and the availability of a therapy that is clearly more effective.

### Randomisation

#### Sequence generation and allocation concealment mechanism

Randomisation will be performed by a centralised trial coordinating centre using an interactive voice response system (IVRS). Authorised staff from the participating centres will submit eligible patients for randomisation. Randomisation will be stratified by centre, gender, age, number of cerebral metastases, presence of extracranial disease and planned WBRT dose using minimisation.

### Blinding

Study participants and treating clinicians will not be blinded to treatment allocation. The centralised personnel assessing MRI scans and neurocognitive function will be blinded to treatment allocation.

### Statistical methods

Efficacy analyses will be conducted on the basis of 'intention to treat' and toxicity analyses will be by treatment received and unadjusted. All comparisons will be 2-tailed with a 5% significance level. The primary endpoint, proportion of patients with distant intracranial failure, will be compared for the two groups using chi-squared or exact tests [16]. Continuous outcomes will be analysed by using t-tests or suitable non-parametric methods if appropriate. The secondary endpoint, time to distant intracranial failure, will be compared for the two groups using the logrank test and Kaplan-Meier curves [16]. Exploratory analyses will be conducted adjusting for prognostic factors using proportional hazards or other suitable regression models. Other time-to-event endpoints will be analysed using similar methods.

#### Subgroup analyses

Deterioration in neurocognitive function and distant intracranial failure will be assessed for the following subgroups: one versus more than one cerebral metastasis; presence of extracranial disease versus none; patients < 65 years of age or ≥65 years of age. Other outcomes assessed for the main study will also be analysed for the subgroups with the acknowledgement that these analyses are exploratory and hypothesis-generating.

### Quality Assurance

#### Radiotherapy treatment delivery

**Figure 2:**
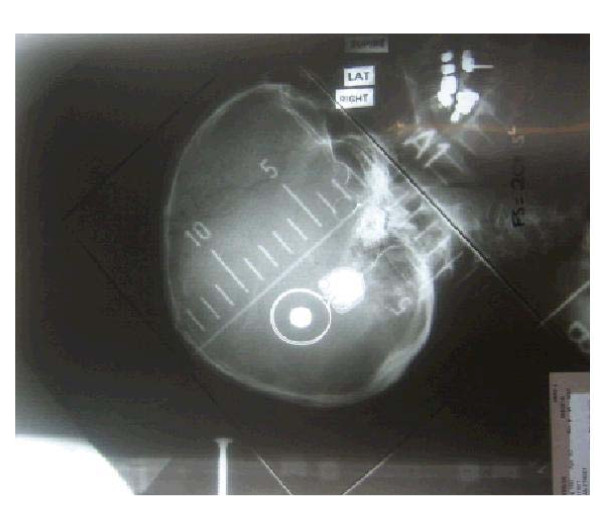
**Plain simulation film showing isocentre on the caudal edge of the field to ensure that there is no divergence through the contralateral eye**. **WBRT Quality Assurance**. *Simulation & Planning*. The patient must be simulated supine, arms by the side, head resting on neck-shape as per department protocol. A personalised immobilisation mask can be used. *Treatment Volume*. The fields must cover the whole brain and include the cerebrum and cerebellum. The superior border of the field must be overshooting the skull by 2 cm to ensure adequate coverage. There must be adequate coverage of the intracranial contents by a margin of 1-2 cm. The caudal border needs to be angled to accomplish this and also avoid the eyes. The pituitary is usually included in the field by default. No effort should be made to cover it if it is not in field.

Technical review for all patients who undergo WBRT will be conducted by the Trans-Tasman Radiation Oncology Group (TROG). Figure 2 provides an example of WBRT simulation, planning and treatment volume. Sites must submit copies of the case history, treatment prescription, treatment administration sheet, dosimetry plans and portal verification films.

#### Radiological audit of MRI scans

At least 25% of the patients will be audited, including the first 5 patients from each centre and a randomly selected patient from each subsequent 5 patients. Auditing will include an evaluation of MRI technique. The audit will be performed by a central radiologist with specialisation in MRI neuroradiology, with reference to a second central radiologist, similarly qualified, at the central radiologist's discretion. This second central radiologist will be blinded to the local and central radiologists' reports.

### Operational considerations

Patients who are randomised to the observation arm of the study and have a recurrence of brain metastases will be able to crossover to WBRT at the treating physician's discretion. All participants will be monitored to death or to the closure of the trial.

## Discussion

### Feasibility

The possibility of conducting this trial was first discussed at a multi-disciplinary melanoma meeting in Sydney in 2004. A previous trial of WBRT for solitary brain metastases from mixed tumour types was abandoned by another Australian/New Zealand study group due to failure to accrue sufficient participants (TROG 98.05) [17]. Given this background and the concern from some investigators about future WBRT trials, a feasibility study was undertaken to assess not only the number of potentially eligible patients but also the willingness of surgical and medical oncologists to have their patients with melanoma randomised to WBRT or not. The trial protocol was presented and critiqued at numerous scientific meetings both nationally and internationally, to multi-disciplinary melanoma treatment centres, and to neuro-oncology groups. There was broad agreement that the trial was needed to answer several fundamental questions and international participation was deemed essential for recruitment success. At present there are three participating countries: Australia, Norway and the United Kingdom. As of the 31st March 2011, 43 patients were randomised with 30 of these completing the neurocognitive function assessment.

#### Registration

This trial is registered with the Australia and New Zealand Clinical Trials Registry (ANZCTR) # ACTRN12607000512426

#### Protocol

A full copy of the current protocol can be requested from the principal investigator: Dr Gerald Fogarty, email: gerald.fogarty@cancer.com.au

## Abbreviations

ANZMTG: Australia and New Zealand Melanoma Trials Group; CTCAE: Common Terminology Criteria for Adverse Events; EORTC: European Organisation for the Research and Treatment of Cancer Data Center; Gy: Gray (unit of radiation); HREC: Human Research Ethics Committee; ICH GCP: International Conference on Harmonisation of Good Clinical Practice; MRI: Magnetic Resonance Imaging; NCF: Neurocognitive function; NHMRC CTC: National Health and Medical Research Council Clinical Trials Centre; QoL: Qualify of Life; SAE: Serious Adverse Event; SRS: Stereotactic Radiosurgery; TMC: Trial Management Committee; TROG: Trans-Tasman Radiation Oncology Group; WBRT: Whole Brain Radiotherapy

## Competing interests

The authors declare that they have no competing interests.

## Authors' contributions

GF conceived of the study, participated in its design, coordination and recruitment and assisted in drafting the manuscript. RLM participated in the design of the study and prepared the first draft of the manuscript. JV participated in the design of the study and reviewed the manuscript. AN participated in the design of the study and reviewed the manuscript. CM participated in the design of the study and reviewed the manuscript. PF participated in the design of the study and contributed to writing of the statistical methods and reviewed the manuscript. AH conceived of the study, and participated in its design and recruitment. GH participated in the design of the study and reviewed the manuscript. BB participated in the design of the study and reviewed the manuscript. HD participated in the design and coordination of the study and reviewed the manuscript. BS is involved in accrual to the study and reviewed the manuscript. JFT conceived of the study, and participated in its design and reviewed the manuscript. All authors read and approved the final manuscript.

## Authors' information

GF, AH and GH are radiation oncologists at Melanoma Institute of Australia (MIA) (formerly the Sydney Melanoma Unit). BB is a radiation oncologist in Brisbane, Queensland and President of the Trans-Tasman Radiation Oncology Group (TROG). RLM is a clinical trialist and executive member of the ANZMTG. JV is a medical oncologist in Sydney, New South Wales, with expertise in neurocognitive function in cancer patients. AN is a medical oncologist in Perth, Western Australia with expertise in quality of life assessment. CM is a neuro-radiologist at a large cancer centre in Melbourne, Victoria. BS is a neurosurgeon at MIA. PF is a biostatistician with expertise in clinical trials design. HD is a behavioural scientist with expertise in neurocognitive function in cancer patients. JFT is a surgical oncologist, chairman of the ANZMTG and executive director of MIA in Sydney, New South Wales.

## Pre-publication history

The pre-publication history for this paper can be accessed here:

http://www.biomedcentral.com/1471-2407/11/142/prepub
